# Tumor Budding Is an Independent Prognostic Factor in Pancreatic Adenocarcinoma and It Positively Correlates with PD-L1 Expression on Tumor Cells

**DOI:** 10.3390/biomedicines10071761

**Published:** 2022-07-21

**Authors:** Rafał Pęksa, Michał Kunc, Piotr Czapiewski, Michał Piątek, Stanisław Hać, Barbara Radecka, Wojciech Biernat

**Affiliations:** 1 Department of Pathomorphology, Medical University of Gdansk, 80-214 Gdansk, Poland; mkunc@gumed.edu.pl (M.K.); biernat@gumed.edu.pl (W.B.); 2 Department of Pathology, Dessau Medical Centre, Auenweg 38, 06847 Dessau-Roßlau, Germany; piotr.czapiewski@med.ovgu.de; 3 Department of Pathology, Medical Faculty, Otto-von-Guericke University Magdeburg, Leipzigerstr. 44, 39120 Magdeburg, Germany; 4 Department of Oncology with Daily Unit, Tadeusz Koszarowski Cancer Center in Opole, Katowicka 66a, 45-061 Opole, Poland; mpiatek@onkologia.opole.pl (M.P.); barbara.s.radecka@gmail.com (B.R.); 5 Department of General Endocrine and Transplant Surgery, Medical University of Gdansk, 80-214 Gdansk, Poland; stanislaw.hac@gumed.edu.pl; 6 Department of Oncology, Institute of Medical Sciences, University of Opole, 45-062 Opole, Poland

**Keywords:** pancreatic cancer, PD-L1, VISTA, budding, thrombosis, inflammation, tumor microenvironment

## Abstract

Pancreatic adenocarcinoma is one of the leading causes of cancer-related death in developed countries. Only 15% of patients are candidates for radical surgery, and adequate prognostication may guide proper postsurgical management. We aimed to retrospectively assess the prognostic significance of the immunohistochemical expression of immune checkpoint receptors (PD-L1 and VISTA), markers of systemic inflammation, thrombosis in the tumor area, and the tumor budding in the group of 107 patients diagnosed with pancreatic adenocarcinoma in a single center. The high expression of PD-L1 on tumor cells (TCs) was associated with worse overall survival (OS, *p* = 0.041, log-rank). On the contrary, high PD-L1 or VISTA on tumor-associated immune cells (TAICs) was correlated with better OS (*p* = 0.006 and *p* = 0.008, respectively, log-rank). The joint status of PD-L1 on TCs and TAICs stratified patients into three prognostic groups. The cases with high-grade budding were characterized by higher PD-L1 expression on TCs (*p* = 0.008) and elevated systemic inflammatory markers. Moreover, budding was identified as the independent prognostic factor in multivariate Cox regression analysis (HR = 2.87; 95% CI = 1.75–4.68; *p* < 0.001). To conclude, the pattern of PD-L1 and VISTA expression was associated with survival in univariate analysis. Tumor budding accurately predicts outcomes in pancreatic cancer and should be incorporated into routine histopathological practice.

## 1. Introduction

Pancreatic adenocarcinoma is one of the leading causes of cancer-related death in developed countries [1]. Most patients are not eligible to undergo curative surgery and are treated palliatively. However, about 15% of patients are candidates for surgical treatment, and the next 15–20% are classified as borderline resectable [2]. The treatment outcomes in these patients are gradually improving, and the currently optimal therapy based on pancreaticoduodenectomy followed by adjuvant chemotherapy with a modified FOLFIRINOX regimen results in median survival of up to 54 months in selected patients [3]. Nevertheless, survival depends on many factors, including perioperative course; comorbidities; age; and, importantly, the intrinsic features of the tumor. Adequate prognostication may guide the proper postsurgical management of the patients.

Histologically, pancreatic adenocarcinoma is usually characterized by prominent desmoplastic stroma, frequent lymphovascular and perineural invasion, necrosis, and thrombosis. These phenomena create a unique microenvironment that is inhabited by various populations of immune cells, including lymphocytes, macrophages, and granulocytes, which interact with cancer cells. The proportions of these cells in the local tumor microenvironment but also peripheral blood may influence outcomes in pancreatic carcinoma [4]. Similarly, proteins expressed by immune cells, especially immune checkpoint receptors and their ligands, e.g., programmed death PD-1/PD-L1 or P-selectin glycoprotein ligand 1 (PSGL-1)/V-domain immunoglobulin suppressor of T cell activation (VISTA), are known predictive and prognostic factors in a plethora of malignancies [5,6,7]. In 2012, a phase I clinical trial on anti-PD-L1 treatment reported no objective responses in pancreatic cancer [8]. Overcoming pancreatic cancer resistance to immunotherapy is a subject of intensive research [9].

Another prognostically important feature of cancer is the pattern of invasion; especially, the presence of tumor budding at the invasive front of the tumor is associated with grim outcomes in gastroenterological cancers [10]. Prominent budding reflects the enhanced mobility and invasiveness of cancer cells. Finally, thrombosis in the tumor area results in hypoxia-altering angiogenesis, tumor proliferation, metabolism, and immune evasion [11].

The current study aims to comprehensively analyze the prognostic significance and reciprocal associations between the expression of immune checkpoint receptors in the tumor microenvironment, the markers of systemic inflammation, thrombosis in the tumor area, and the grade of tumor budding in pancreatic carcinoma.

## 2. Materials and Methods

### 2.1. Study Group

The inclusion criteria were a diagnosis of pancreatic ductal carcinoma treated with radical surgery, and available archival tissue material. The exclusion criteria were a lack of baseline clinicopathological data and the death of the patient due to perioperative complications. Eligible patients were identified in our central database using the MedStream Designer tool (Transition Technologies, Łódź, Poland). We retrospectively enrolled a consecutive series of 107 (n = 107) patients who underwent radical pancreatectomy from 2009 to 2018 in our center and met the inclusion criteria. Anonymized basic clinicopathological data (tumor location, grade, stage according to pTNM, surgical margins, the presence of perineural invasion (PNI), lymphovascular invasion (LVI), comorbidities, complete blood count (CBC), adjuvant treatment, date of relapse, and date of death were retrieved from the database. Preoperative neutrophil-to-lymphocyte ratio (NLR), platelet-to-lymphocyte ratio (PLR), and monocyte-to-lymphocyte ratio (MLR) were calculated for each patient.

The study was conducted in accordance with the Declaration of Helsinki and approved by the Bioethical Committee of the Medical University of Gdańsk (approval No. NKBBN/129/2020-2021).

### 2.2. Histopathology and Immunohistochemistry

Archival formalin-fixed paraffin-embedded (FFPE) tissue blocks and hematoxylin and eosin stained microscopic slides were retrieved. Subsequently, a reevaluation of primary slides was performed by two pathologists blinded to clinical data. During this step, the number of thrombotic blood vessels in the tumor center and periphery was counted, similarly to the grade of tumor budding according to the guidelines established for colorectal carcinoma [12]. Tissue microarrays (TMAs) were constructed with Manual Tissue Arrayer MTA-1 (Beecher Instruments, Inc., Sun Praire, WI, USA) using 1.5 mm core needles. In each case, three representative areas containing cancer tissue and stroma were selected.

Obtained TMAs were stained with the antibodies against PD-L1 (clone 22C3, 1:50 dilution, DAKO, Glostrup, Denmark) and VISTA (clone D5L5T, 1:300 dilution, Cell Signaling, Danvers, MA, USA). The expression of immune checkpoint receptors was assessed separately in tumor cells (TCs) and tumor-associated immune cells (TAICs) using a proportion score (the percentage of positively staining cells of each type). Histologically normal tonsil and placenta were used for the positive control. The omission of the primary antibody was used as a negative control. Only a complete membranous reaction in TCs was considered positive, whereas both membranous and cytoplasmatic reactions in the TAICs were counted as positive.

### 2.3. Statistics

Statistical analyses were performed with the use of Statistica 13 (Tibco Software Inc., Palo Alto, CA, USA) and R Statistical Environment [13]. Associations between analyzed markers and clinicopathological parameters were analyzed with the chi-square test, Fisher’s exact test, and Wilcoxon test when applicable. Boxplots were plotted using the “ggplot2” package [14]. A *p*-value < 0.05 was considered as significant.

The optimal cut-off values of semi-quantitative and quantitative variables for survival analyses were identified with the use of the surv cutpoint function from the “survminer” package [15]. Kaplan–Meier curves were plotted using the “survminer” and “ggsci” packages [15,16]. Uni- and multi-variate Cox regression analyses were performed to estimate hazard ratios (HRs). In multivariate analysis, the stepwise selection with a *p*-value to enter <0.15 and a *p*-value to remove <0.05 was performed to eliminate insignificant variables.

## 3. Results

### 3.1. Characteristics of the Study Group

The median age of patients was 66 years (IQR: 61–74). The sex distribution was almost equal. The vast majority of patients presented with pT2 and pT3 tumors (88%) and nodal metastases (79%). The median follow-up was 438 days (IQR: 270–698). In the follow-up period, 93 patients died (87%). One-year OS was 60%, and 2-year OS was 28.6%. The summary of other clinicopathological features is presented in Table 1.

### 3.2. Expression of Immune Checkpoint Receptors in Pancreatic Cancer Cells and Immune Cells

**Figure 1:**
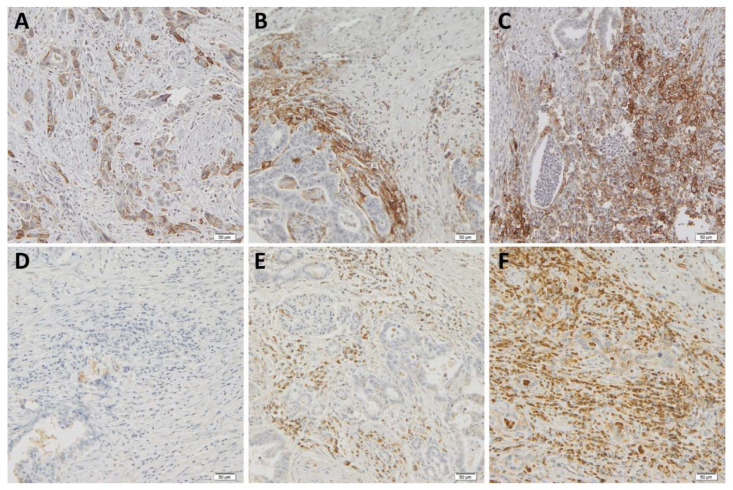
Representative examples of immunohistochemical stainings. (**A**) Intense, membranous PD-L1 reaction in pancreatic carcinoma cells with prominent budding; (**B**) high expression of PD-L1 restricted to TAICs and lack of reaction in TCs; (**C**) PD-L1 staining visible mainly in TAICs and in some TCs; (**D**) negative VISTA staining reaction in both TAICs and TCs; and (**E**,**F**) high expression of VISTA in the majority of TAICs and lack of reaction in TCs.

The optimal cut-off values for immune checkpoint receptor expression in terms of prognostication identified with the use of the surv_cutpoint() function were 3% of PD-L1-positive TAICs, 1% of PD-L1-positive TCs, and 4% of VISTA-positive TAICs. According to these thresholds, the cases were divided into low- and high-expression groups. The expression of PD-L1 in >3% of TAICs was noted in 47 cases (44%), whereas 41 tumors (38%) displayed PD-L1 in >1% of TCs. There was no association between the expression of PD-L1 on TAICs and TCs (*p* = 0.425, chi-square). VISTA was consistently negative on TCs in all cases, whereas 69 cases showed >4% of VISTA-positive TAICs (68%; evaluable in 101 cases). The expression of VISTA and PD-L1 on TAICs was positively correlated (*p* = 0.004, chi-square), but there was no association between VISTA and PD-L1 on TCs (*p* = 0.646, chi-square). The representative examples of PD-L1 and VISTA staining are shown in Figure 1.

Taking into consideration basic clinicopathological parameters, the high expression of PD-L1 on TAICs was less common in tumors with LVI (39.8% vs. 71.4%, *p* = 0.026, chi-square). High WHO grade tumors (G3) tended to have high PD-L1 expression on TCs when compared to G1 and G2 cases (*p* < 0.001, chi-square). We did not find any other relationship with clinicopathological parameters, including tumor stage, patients’ sex or age, perineural invasion, and immune checkpoint receptor expression.

### 3.3. Immune Checkpoint Receptors and Markers of Systemic Inflammation

**Figure 2:**
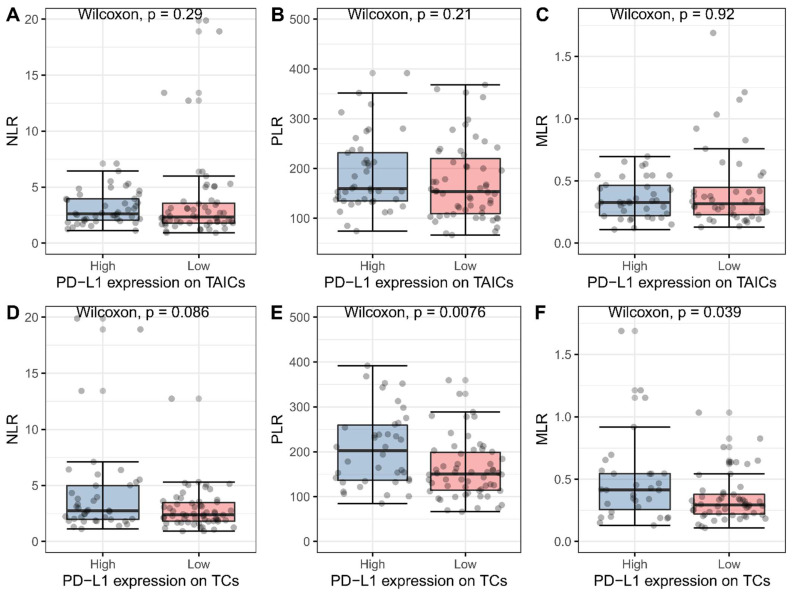
Associations between markers of systemic inflammation and PD-L1 expression on tumor-associated immune cells (TAICs) (**A**–**C**) and tumor cells (TCs) (**D**–**F**). Blue and red boxes represent high and low expression of PD-L1, respectively. Grey dots represent individual measures.

The median values of markers of systemic inflammation (NLR, PLR, and MLR) are given in Table 2. The values of pre-operative PLR and MLR and post-operative PLR were associated with PD-L1 expression on TCs but not PD-L1 or VISTA on TAICs (Figure 2).

### 3.4. Immune Checkpoint Receptors and Thrombosis

The presence of thrombosis was assessed separately inside the tumor and at the tumor periphery in blood vessels of various calibers. In general, thrombosis was observed inside the tumor in 62 tumors (58%) and at the tumor periphery in 37 tumors (35%). The presence of thrombosis in the tumor mass was associated with a lower expression of PD-L1 on TAICs (Table 3). The expression of VISTA on TAICs was not associated with thrombosis. There was no relationship between the caliber and the number of the thrombotic vessels (data not presented).

### 3.5. Immune Checkpoint Receptors and Budding

**Figure 3:**
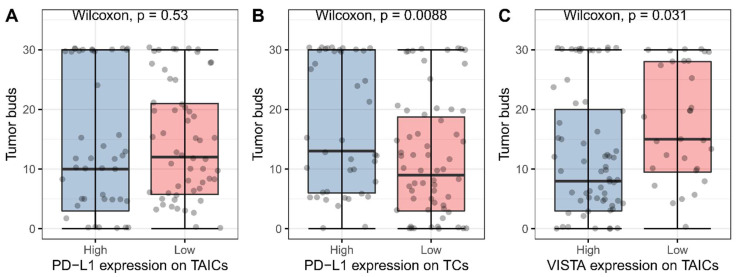
Association between PD-L1 and VISTA expression and the number of tumor buds (**A**–**C**). Blue and red boxes represent high and low expression of immune checkpoint receptors, respectively. Grey dots represent individual measures.

The median number of tumor buds was 11 (range 0–30). The cases with PD-L1-positive TCs and VISTA-positive TAICs were characterized by a higher number of tumor buds (*p* = 0.008 and *p* = 0.031, respectively, Wilcoxon test, Figure 3). No such association was noted for PD-L1 on TAICs (*p* = 0.534, Wilcoxon test). Additionally, we noted that tumors with PD-L1-positive TCs show a bimodal distribution of a number of buds. The PD-L1-positive cases with high-grade budding presented with a higher WHO grade (*p* = 0.031, chi-square), lack of mucus production (*p* = 0.02, chi-square), and a higher percentage of PD-L1-positive TCs (*p* = 0.064, Wilcoxon test).

### 3.6. Univariate Survival Analysis

#### Immune Checkpoint Receptors

**Figure 4:**
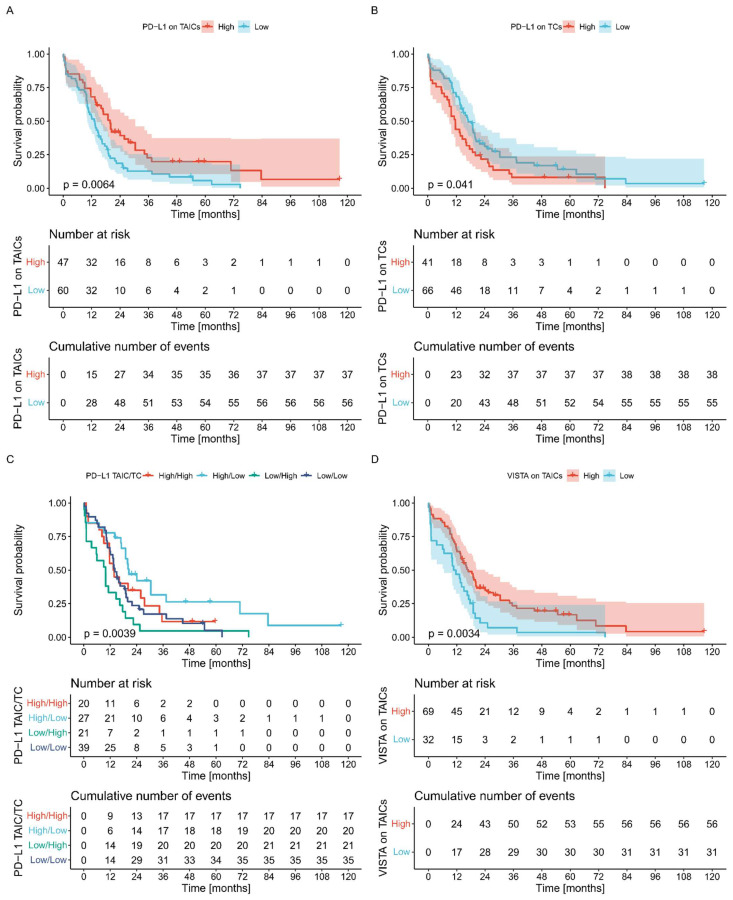
The Kaplan–Meier plots for OS stratified by PD-L1 on TAICs (**A**), PD-L1 on TCs (**B**), combined PD-L1 on TAICs/TCs (**C**), and VISTA on TAICs expression (**D**) in pancreatic adenocarcinoma. *p*-values were calculated with log-rank.

High expression of PD-L1 on TCs was associated with worse OS (*p* = 0.041, log-rank) (Figure 4). On the other hand, high PD-L1 or VISTA on TAICs was correlated with better OS (*p* = 0.006 and *p* = 0.008, respectively, log-rank). Interestingly, the joint status of PD-L1 on TCs and TAICs stratified patients into three prognostic groups. Tumors characterized by low PD-L1 on TAICs and high PD-L1 on TCs showed the worst outcomes, whereas high PD-L1 on TAICs and low PD-L1 on TCs were associated with the best survival. Other combinations displayed intermediate survival (Figure 4).

### 3.7. Other Analyzed Variables

**Figure 5:**
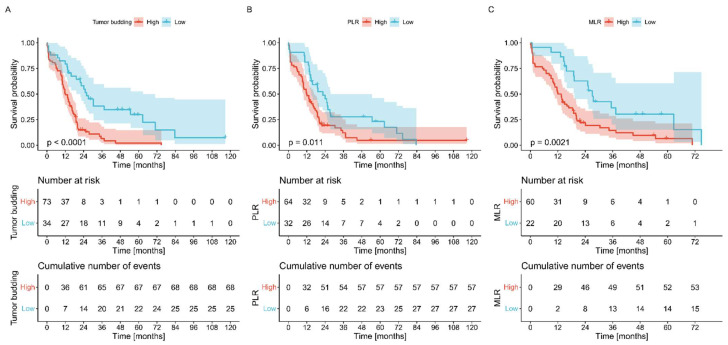
The Kaplan–Meier plots were stratified by budding grade (**A**), PLR values (**B**), and MLR values (**C**). *p*-values were calculated with log-rank.

High preoperative levels of markers of systemic inflammation, especially PLR and MLR, were generally associated with poor OS (Figure 5). Postoperative values did not show any significant relationship with survival. We did not observe any influence of thrombosis in the tumor area on survival in patients with pancreatic cancer. On the other hand, high-grade budding indicated grim outcomes (Figure 5).

### 3.8. Multivariate Survival Analysis

In the multivariate Cox regression with stepwise regression to eliminate insignificant variables at *p* < 0.05, the only independent factor identified predicting survival in our cohort was tumor budding (Table 4). Nevertheless, in the alternative multivariable model incorporating all variables from univariate analysis with *p* < 0.05, the ratio of affected to non-affected lymph nodes (HR = 2.33, 95% CI = 1.27–4.77, and *p* = 0.007), PD-L1 on TCs (HR = 1.83, 95% CI = 1.05–3.16, and *p* = 0.032), and PLR (HR = 2.16, 95% CI = 1.23–3.83) were recognized as statistically significant prognostic factors.

## 4. Discussion

In the current study, we aimed to identify the relationships between immune checkpoint receptor expression and several clinicopathological markers that may influence the tumor microenvironment: systemic inflammation (NLR, PLR, and MLR), tumor thrombosis, and tumor budding. Moreover, we performed survival analysis to investigate their prognostic capabilities. This is the first study to comprehensively assess these markers. We demonstrated that high levels of immune checkpoint receptors on TAICs in pancreatic cancer correlate with better OS, whereas high PD-L1 expression on TCs is associated with unfavorable outcomes. A combined assessment of PD-L1 on TCs and TAICs stratified patients into three risk groups. Pancreatic carcinomas with high-grade budding tended to express PD-L1 on TCs, probably evading an immune response and enhancing their biological aggressiveness. Similarly, systemic inflammation markers positively correlated with PD-L1 on TCs, and their higher levels indicated worse OS. Finally, the presence of thrombotic blood vessels correlated with the higher number of PD-L1-positive TAICs, suggesting the possible role of hypoxia in modulating the condition of the tumor microenvironment. Table 5 summarizes the correlations between the analyzed biomarkers.

Pancreatic cancer is frequently characterized by an immunologically “cold” microenvironment due to desmoplastic stroma limiting the T cell infiltration [17]. Various preclinical models were proposed to study the response to immune therapy in pancreatic cancer. Interestingly, patient-derived xenografts (PDX) models might be not suitable for this purpose since they are implanted in immunocompromised mice and lack the donor immune system [17]. Other animal models including syngenic or genetically-engineered mouse models at least partially recapitulate the immunosuppressive environment of human pancreatic malignancies. These tumors are frequently deprived of effector T-cells but are infiltrated by immunosuppressive cells and are rather refractory to immune checkpoint inhibitors. Animal models demonstrate that combining chemotherapy with the targeting of various elements of the immune microenvironment may enhance the treatment efficacy [18,19]. A very recent study demonstrated that a combination of nivolumab (anti-PD-1 agent) and gemcitabine in metastatic pancreatic carcinoma patients improved the 1-year OS rate compared to a historical chemotherapy cohort [20]. Importantly, the authors emphasized that the assessment of pre-treatment biomarkers, e.g., the circulating T-follicular cells level, may help to choose an optimal, personalized immune therapy regimen [20].

Early studies suggested that the down-regulation of VISTA may contribute to immune evasion in pancreatic cancer [21]. When compared to melanoma, pancreatic cancer displays a significantly higher number of VISTA-positive cells [7]. We and others observed a positive correlation between PD-L1 and VISTA expression, but these two immune checkpoint receptors are not redundant and represent two distinct potential therapeutic targets [22]. The feasibility of VISTA targeting in clinical practice is yet to be established, but anti-VISTA antibody treatment reduced the metastatic burden in the murine model of pancreatic cancer liver metastases [23]. Anti-PD-L1 monotherapy in pancreatic cancer is not very beneficial clinically [9]. Likewise, the overexpression of VISTA in pancreatic cancer is usually accompanied by additional myeloid or metabolic immunosuppressive mechanisms, which may diminish the benefits of anti-VISTA therapy [24]. Thus, cotargeting the PD-1/PD-L1 axis and VISTA is a potential option to enhance the response to therapy in selected cases [25].

Liu et al. demonstrated that VISTA expression is mainly restricted to immune cells, predominantly CD68^+^ macrophages, whereas TCs show no or minimal expression [26], which is consistent with our results. Nevertheless, a recent study reported VISTA expression in 25.6% of TCs and 38.1% of immune cells in pancreatic carcinoma [23]. VISTA expressed in TCs but not on immune cells was associated with a positive prognosis. This study utilized the same antibody as ours (D5L5T) but with lower dilution (1:25 vs. 1:300), which may explain the discrepancy in TCs staining results. Another study by Popp et al. identified VISTA-positive TAICs in 46.1% of cases with a trend toward better survival, but this finding was not statistically significant [27]. However, the authors used a different antibody clone and a lower positivity threshold than in our study.

The prognostic value of PD-L1 in pancreatic adenocarcinoma was investigated in numerous studies. A meta-analysis based on 9 studies with 993 patients showed that elevated PD-L1 expression was related to poor OS and cancer-specific survival, nodal metastases, advanced T stage, and high histological grade [28]. Nevertheless, few studies reported better survival in cancers expressing high PD-L1 [29]. However, these studies did not discriminate between PD-L1 expression on TAICs or TCs, which seems to be a crucial issue. A recent multiplex immunofluorescence study reported that a “constitutive” immune phenotype characterized by PD-L1 expression on TCs accompanied by a lack of PD-L1-expressing TAICs (immune desert) indicates the worst outcomes in pancreatic cancer [30]. On the other hand, the “combined” pattern with PD-L1-negative TCs and PD-L1-positive immune cells displayed a favorable prognosis. These results are in line with our findings and underscore the importance of the separate PD-L1 expression on TCs and immune cells since the pattern of expression carries important prognostic information.

Epithelial-mesenchymal transition (EMT) is one of the mechanisms enhancing invasiveness and metastatic potential in solid tumors. The activation of EMT leads to increased mobility and loss of cohesion between cancer cells. Importantly, EMT may induce immune evasion contributing to cancer progression [31]. However, the relationship between EMT and PD-L1 expression is reciprocal since PD-L1 expression boosts the cell viability and mobility in esophageal carcinoma cell lines [32]. Our study demonstrated the positive correlation between high-grade tumor budding and PD-L1 expression on TCs. This finding is supported by a recent study by Sadozai et al., which demonstrated that extensive tumor budding is accompanied by diminished anti-tumor immunity and higher B7-H3 (CD276) expression [33]. In colorectal cancer, high-grade tumor budding is associated with an up-regulation of negative regulatory immune checkpoints and immune evasion [34]. We and others identified tumor budding as the independent prognostic factor in pancreatic cancer [35,36]. Additionally, there is a high concordance between pathologists in the assessment of tumor budding in pancreatic cancer [36]. Taking into consideration the high reproducibility and prognostic power of this characteristic, it has significant potential to be incorporated into routine histopathological practice [37].

Local tumor microenvironment and systemic immune or inflammatory response seem to influence each other in cancer patients [38]. Xiang et al. demonstrated that high NLR was associated with worse OS and decreased CD8^+^/CD28^−^ and CD4^+^/CD25^+^ cell subsets in pancreatic cancer settings [39]. Another study showed that soluble PD-L1 (sPD-L1) and NLR were independent prognostic factors in pancreatic carcinoma [40]. In one study, PD-L1 mRNA expression in plasma-derived microvesicles was not related to NLR and PLR values [41]. Nevertheless, in the current study, we found a positive correlation between MLR and PLR levels and PD-L1 expression on TCs. A similar relationship has been described for example in bladder carcinoma [42], indicating that systemic inflammation may promote immune evasion in the tumor microenvironment. In non-small cell lung carcinoma, NLR was identified as a potential predictive marker for treatment with second-line pembrolizumab [43]. Thus, markers of systemic inflammation should be investigated as predictive markers for immunotherapy response in pancreatic cancer.

Finally, we observed an association between the presence of thrombosis and high PD-L1 expression on TAICs. Thrombosis results in local hypoxia and hypoglycemia, influencing the tumor microenvironment. Hypoxic TAICs change their metabolism and may switch to ketone bodies as the energy source [44]. Many studies to date have demonstrated that hypoxia induces PD-L1 expression on TCs in various malignancies, enabling immune escape [45,46]. Less is known about hypoxia and immune checkpoint receptors-expressing TAICs. Nevertheless, one study demonstrated that hypoxia enhances the effector responses of CD8+ lymphocytes to persistent antigens and increases immune checkpoint CTLA-4 expression [47]. Under a hypoxic environment, various immunosuppressive cells, including myeloid suppressor cells, macrophages, and dendritic cells, up-regulate PD-L1, promoting cancer immune evasion [45]. Early studies suggest that inhibiting thrombosis and alleviating hypoxia with anticoagulants may enhance the response to immune checkpoint inhibitors [11,48]. It is yet to be determined if the use of anticoagulants may enhance immune therapy efficacy in patients with pancreatic cancer.

The main limitation is the retrospective nature of the study, which is based on single-center data. We failed to demonstrate the prognostic value of the stage in our cohort, which is most likely a consequence of the relatively small sample size, the high heterogeneity of the cohort, and probably a high number of patients with various comorbidities. We were also unable to analyze the impact of adjuvant treatment on outcomes since many patients underwent treatment in other facilities and these data were incomplete. Similarly, we were unable to retrieve some crucial baseline data (the ECOG performance status scale, comorbidities, and surgical complications). Thus, due to the lack of this information, the results of the current study should be interpreted with caution. Moreover, the results are mainly based on immunohistochemical stains of TMAs, which contain a small sample of the tumor and may not adequately reflect potential intratumor heterogeneity.

## 5. Conclusions

Effective treatment and outcome prediction in pancreatic cancer remain a challenge. To improve the management of this malignancy, we need to investigate the complex relationships in the tumor microenvironment. In the current study, we demonstrated associations between high-grade budding and systemic inflammatory markers and PD-L1 expression on TCs, as well as local thrombosis and PD-L1 expression on TAICs. The pattern of PD-L1 and VISTA expression was associated with survival in univariate analysis. Future studies on in vivo models should investigate the mechanistic role of PD-L1- or VISTA-expressing TAICs in the biology of pancreatic cancer. Tumor budding accurately predicts outcomes in pancreatic cancer and should be incorporated into routine histopathological practice. The alternative model incorporated the status of lymph nodes, PD-L1 on TCs, and PLR. Future studies should investigate the potential predictive significance of these factors on immunotherapy in pancreatic carcinoma.

## Figures and Tables

**Table 1:** Summary of basic clinicopathological data.

Feature		N	%
Sex	Male	51	48%
	Female	56	52%
Tumor location	Head	89	83%
	Corpus/Tail	15	14%
	Other	3	3%
Grade (WHO)	1	16	15%
	2	44	41%
	3	47	44%
pT	1	10	9%
	2	59	55%
	3	37	35%
	4	1	1%
pN	0	23	21%
	1	49	46%
	2	35	33%
R	0	49	46%
	1	43	40%
	2	15	14%
PNI	0	8	7%
	1	99	93%
LVI	0	14	13%
	1	93	87%
Death	0	14	13%
	1	93	87%

**Table 2:** Median and interquartile (IQR) values for markers of systemic inflammation: neutrophil-to-lymphocyte ratio (NLR), platelet-to-lymphocyte ratio (PLR), and monocyte-to-lymphocyte ratio (MLR).

	Marker	Median	IQR
Prior to sugery	NLR	2.49	1.86–3.81
	PLR	155	124–229
	MLR	0.33	0.23–0.47
After surgery	NLR	11.7	7.66–18.54
	PLR	241	149–351
	MLR	0.65	0.36–0.71
Ratio prior/after	NLR	0.24	0.15–0.40
	PLR	0.76	0.55–1.06
	MLR	0.63	0.42–0.97

**Table 3:** Associations between the presence of thrombosis in various compartments and PD-L1 expression on TAICs and TCs.

Thrombosis		PD-L1 on TAICs		*p*	PD-L1 on TCs		*p*
		Low	High		Low	High	
Tumor	0	20 (33)	25 (53)	0.039	27 (41)	18 (44)	0.760
	1	40 (67)	22 (47)		39 (59)	23 (56)	
Periphery	0	35 (58)	35 (74)	0.082	43 (65)	27 (66)	0.941
	1	25 (42)	12 (26)		23 (35)	14 (34)	
Any	0	6 (10)	20 (43)	<0.001	15 (23)	11 (27)	0.630
	1	54 (90)	27 (57)		51 (77)	30 (73)	

**Table 4:** Uni- and multi-variate proportional hazard Cox regression analysis of prognostic markers in pancreatic cancer.

Feature	Univariate			Multivariate		
	HR	95% CI	*p*	HR	95% CI	*p*
T	0.989	0.64–1.52	0.958			
N	1.33	0.80–2.22	0.261			
N ratio	1.79	1.06–3.03	0.028			
Stage	1.09	0.70–1.71	0.688			
Grade	1.65	1.09–2.50	0.017			
Buds (≤5 vs. >5)	2.87	1.75–4.68	<0.001	2.87	1.75–4.68	<0.001
NLR	1.97	0.94–4.10	0.071			
PLR	1.82	1.14–2.92	0.011			
MLR	2.42	1.35–4.33	0.002			
PD-L1 on TAICs	0.56	0.37–0.85	0.007			
PD-L1 on TCs	1.53	1.01–2.33	0.042			
VISTA on TAICs	0.51	0.33–0.81	0.004			
Tumor thrombosis	1.08	0.71–1.63	0.710			
Peripheral thrombosis	0.96	0.63–1.49	0.883			
Any thrombosis	0.77	0.47–1.26	0.307			

**Table 5:** The summary of correlation between analyzed parameters. Green depicts positive correlation, red depicts negative correlation, and grey means no significant correlation.

Features							
	Budding						
PD-L1 on TCs		PD-L1 on TCs					
PD-L1 on TAICs			PD-L1 on TAICs				
VISTA on TAICs				VISTA on TAICs			
PLR					PLR		
MLR						MLR	
Tumor thrombosis							Tumor thrombosis
Overall survival							

## Data Availability

Additional information is available from corresponding author upon reasonable request.
